# Different Non-cage Housing Systems Alter Duodenal and Cecal Microbiota Composition in Shendan Chickens

**DOI:** 10.3389/fvets.2021.728538

**Published:** 2021-10-06

**Authors:** Yi Wan, Ruiyu Ma, Hongyi Zhang, Ling Li, Lilong Chai, Renrong Qi, Wei Liu, Junying Li, Yan Li, Kai Zhan

**Affiliations:** ^1^Anhui Key Laboratory of Livestock and Poultry Product Safety Engineering, Institute of Animal Husbandry and Veterinary Medicine, Anhui Academy of Agriculture Science, Hefei, China; ^2^Hubei Shendan Health Food Co., Ltd., Anlu, China; ^3^Department of Poultry Science, University of Georgia, Athens, GA, United States

**Keywords:** non-cage housing system, duodenum, cecum, microbiota, chicken

## Abstract

Housing systems are among the most important non-genetic factors affecting hen production performance and intestinal microbes. With increased interest in animal welfare, cage-free laying hen housing systems have become common, providing behavioral freedom and health benefits. The present study aimed to compare the effects of plastic net housing system (NRS) and floor litter housing system (LRS) on the composition and function of the duodenal and cecal microbiota in Shendan chicken, one of the most popular laying hen strains in China. The associations between the differential microbiota abundance and production traits and intestinal morphological parameters were determined. Compared with the LRS, the NRS improved the laying rate (*p* < 0.05) and increased the villus height (VH) of the duodenum (*p* < 0.05) and the VH-to-crypt depth ratio (VCR) of the cecum (*p* < 0.05). Alpha diversity analysis showed that LRS chickens had a significantly higher diversity and richness than NRS chickens. Beta diversity analysis demonstrated differences in the microbiota composition based on housing systems. Within the cecum, Proteobacteria and Kiritimatiellaeota were significantly more abundant in the LRS than in the NRS (*p* < 0.05), while Bacteroidetes were significantly less abundant in the LRS (*p* < 0.05). *Phascolarctobacterium* and *Ruminococcaceae_UCG-005* were significantly less abundant in the LRS (*p* < 0.05) compare to the NRS. Within the duodenum, *Lactobacillus* was significantly less abundant in the LRS (*p* < 0.05) than in the NRS, while *Pseudomonas* was significantly more abundant in the LRS (*p* < 0.05). Cecal *Phascolarctobacterium* and *Ruminococcaceae_UCG-005* were significantly positively correlated with eggshell strength (*R* = 0.608, *p* < 0.01) and egg weight (*R* = 0.526, *p* < 0.05), respectively. Duodenal *Lactobacillus* was significantly positively correlated with VH and VCR (*R* = 0.548 and 0.565, *p* < 0.05), while *Pseudomonas* was significantly negatively correlated with the Haugh unit (*R* = −0.550, *p* < 0.05). In conclusion, there are differences in the cecal and duodenal microbiota compositions of Shendan laying hens reared in different non-cage housing systems, and the NRS was superior to the LRS in improving the laying performance and intestinal morphology and microecological environment.

## Introduction

The microbes in poultry intestines play an essential role in feed conversion (1), nutrient digestion and absorption (2), host protection against pathogens (3), and the maintenance of intestinal physiological balance (4) by affecting the intestinal structure and modulating the function of the digestive and immune systems. The duodenum is crucial for food digestion and absorbs most glucose and other nutrients, with the phyla Proteobacteria and Firmicutes being predominant (5, 6). The cecum mainly ferments complex carbohydrates and has a greater ability to absorb sugars actively at low concentrations, with the phyla Bacteroidetes and Firmicutes being predominant (6, 7). However, the diversity and community structure of gut microbes in chickens are influenced by many factors, such as dietary changes, geographical locations, growth phases, and rearing conditions. Feeding broilers with fructooligosaccharide enhanced the growth of *Bifidobacterium* and *Lactobacillus* but inhibited *Escherichia coli* in the small intestinal and cecal digesta (8). Variations in microbe composition and diversity were found among populations of chicken cecal bacteria from five locations in Tibet (9). The ileum and cecum developed their own unique bacterial community during different growth periods as the broilers matured (10). Dysbiosis of the intestinal microbiota could lead to impaired digestion and immunity, which causes an increase in susceptibility to pathogens and results in reduced growth performance and health status.

Although extensive studies concerning the factors that affect bacterial microbes in the gut of chickens have been conducted, little is known about the effects of housing systems on intestinal bacterial communities and functions in chickens. The abundance of *Faecalibacterium, Ruminococcaceae*, and *Helicobacter* in the gut was found to be significantly greater in Lohmann hens raised in cage rearing systems than in those raised in free-range systems (11). A higher abundance of cecal microbiota associated with functions involved in amino acid and glycan metabolic pathways was observed in free-range Dagu chickens than in cage reared chickens (12). With the increased interest in animal welfare, poultry housing systems have been a concern for the last decade, and conventional cages have been gradually replaced by non-cage systems (13). Non-cage housing systems are perceived as being more respectful to animal welfare than cage housing systems which could allow behavioral freedom and promote eco-friendliness (14). There are two primary non-cage housing systems for laying hens in China: the plastic net housing system (NRS) and floor litter housing system (LRS). The NRS comprises perforated plastic nets isolated above the ground, which keep hens away from excreta and could maintain good environmental hygiene. The LRS uses various litters, such as wood shavings, straw or rice husks; it puts hens directly in contact with feces and requires more floor space. Gut microbiota is a good indicator of variations innutrient digestion and absorption capacity of laying hens, which could be affected by environmental condition changes (15). Therefore, it is of great significance to investigate the intestinal microbiota or morphology of hens raised in different non-cage housing systems with the ban on housing hens in conventional cages.

The Shendan chicken, which originated in North China, is one of the most popular local laying hen breeds in China. Shendan chickens are native to Anlu City, Hubei Province, China, and are characterized by its black plumage and blue-colored eggshells. Whether the formation of the gut microflora is affected by different non-cage housing systems and its association with production performance and intestinal health in Shendan chickens are unknown. The present study used the 16S rRNA sequencing approach to analyze the changes in species abundance and diversity of intestinal microbes in Shendan chickens under different non-cage housing systems and to explore their association with production traits and intestinal morphology to help us better master Shendan chicken management practices. The results of this study will provide a better understanding of the effects of non-cage housing systems on the intestinal microbial ecology of Shendan chickens and support poultry production and welfare.

## Materials and Methods

### Animal Feeding and Management

This study was conducted at Hubei Shendan Health Food Co., Ltd. (Anlu, China). A total of 1,200 30-week-old Shendan laying hens with similar body weights (1,295.20 g ± 106.54) raised in cages were selected and randomly divided into NRS and LRS groups. Each group included 5 replicate pens with 120 birds in each replicate. Birds in the NRS treatment group were raised indoors on a perforated plastic floor; the feces that dropped onto the belt under the plastic floor and were removed daily. Birds in the LRS treatment group were raised indoors on a floor covered with wood shavings that were cleaned every 2 weeks. Each replicate pen in both groups had the same indoor stocking density (4.4 birds/m^2^) and had an adjacent outdoor free-range paddock area measuring 8 × 6 m (2.5 birds/m^2^). There was a plurality of nest boxes in indoor houses for hens to lay eggs. The outside paddock, which was used as an activity field, was separated from surrounding areas by wire fences, and the separation of replicates was achieved using fish nets. Feeders and plastic water tanks were located in both the indoor and paddock areas. There were also perches available for the chickens to rest upon. A preliminary trial was conducted for 2 weeks, and the formal experiment was performed from weeks 32 to 40. The poultry houses with the two housing systems were close to one another.

### Production Performance

Eggs were collected and counted every day to calculate the laying rate. Twenty eggs were randomly sampled from each replicate for egg quality analysis every 2 weeks. All eggs were kept in the same storage room, and egg quality measurements were completed on the day of collection. Egg weight was measured using an electronic scale with an accuracy of 0.01 g. Shell strength was measured with an eggshell force gauge (EGG-0503, Robotmation Co., Ltd., Tokyo, Japan). Haugh units were measured using an automatic egg multitester (EMT-5200, Robotmation Co., Ltd., Tokyo, Japan).

### Measurements of Intestinal Morphology

At 40 weeks of age, four birds per replicate in each experimental group were randomly selected for intestinal morphological observation. Birds were sacrificed by CO_2_ suffocation. One-centimeter sections from the duodenum and cecum were carefully removed and immediately fixed in 10% formaldehyde phosphate buffer for the microscopic assessment of mucosal morphology. For morphometric analysis, segments were fixed in a 10% neutral buffered formalin solution for 24 h. Intestinal samples were then excised, dehydrated in a tissue processing machine (Leica Microsystem K. K., Tokyo, Japan) and embedded in paraffin wax. Four-millimeter sections were cut from each sample, fixed onto slides, stained with hematoxylin and eosin, mounted and examined under a light microscope. Stained slides were observed under a Motic BA210, and visual measurements of villus height (VH) and crypt depth (CD) were performed at a magnification of 10 × (objective lens) with imaging software (Motic Image Plus 2.0^ML^ Soft, Motic China Group Co., Ltd., Xiamen, China). VH was measured from the crypt-villus junction to the brush border at the tip. CD was calculated at the level of the basement membranes of opposing crypt epithelial cells. The ratio of VH to crypt depth (VCR) was calculated.

### Sample Collection

At 40 weeks of age, ten birds from each group (two from each replicate) were immediately dissected using sterile scissors to aseptically remove the intestines from the abdominal cavity, and the contents of the duodenum and cecum were gently squeezed into 2 mL cryopreservation tubes and stored immediately at −80°C for further analysis.

### Bacterial DNA Extraction and 16S rRNA Sequencing

Microbial DNA was extracted using HiPure Stool DNA Kits (Magen, Guangzhou, China) according to the manufacturer's protocols. The concentration and integrity of the DNA was verified using a NanoDrop™ 2000 spectrophotometer (Thermo Scientific, MA, USA) and agarose gel electrophoresis. The DNA samples were stored at −80°C until processing for amplification. To construct 16S rDNA sequencing libraries, the V3-V4 regions of the 16S rDNA gene was amplified from the DNA samples by PCR using the universal primers 341 F and 806 R (341F: CCTACGGGNGGCWGCAG; 806R: GGACTACHVGGGTATCTAAT). The negative control is water, and the positive control is the sample with stable amplification in the previous experiment. PCR reaction was carried out in a 50 μL reaction volume with TransGen High-Fidelity PCR SuperMix (TransGen Biotech, Beijing, China), 0.2 μM forward and reverse primers, and 5 ng template DNA. The PCR condition was as follows: 95°C for 2 min, followed by 35 cycles of 95°C for 30 s, 60°C for 45 s, and 72°C for 90 s, with a final extension of 72°C for 10 min. Amplicons were evaluated with 2% agarose gels and purified using the AxyPrep DNA Gel Extraction Kit (Axygen Biosciences, Union City, CA, USA) according to the manufacturer's instructions. Sequencing libraries were generated using the SMRTbell TM Template Prep Kit (PacBio, Menlo Park, CA, USA) following the manufacturer's recommendation. The library quality was assessed with Qubit 3.0 Fluorometer (Thermo Fisher Scientific, USA) and FEMTO pulse system (Agilent Technologies, Santa Clara, CA, USA). The libraries were sequenced on an Illumina HiSeq 2500 platform.

### Bioinformatics Analysis

Paired-end clean reads were merged as raw tags using FLASH (16) (version 1.2.11) with a minimum overlap of 10 bp and mismatch error rates of 2%. Quality filtering of the raw tags was performed under specific filtering conditions (17) according to obtain high-quality clean tags. The filtering conditions are as follows: raw tags were broken from the first low quality base site where the number of bases in the continuous low-quality value (the default quality threshold is ≤ 3) reaches the set length (the default length is 3 bp), and then tags with a continuous high-quality base length <75% of the tag length were filtered. The obtained clean tags were clustered into operational taxonomic units (OTUs) of ≥97% similarity using the UPARSE (18) (version 9.2.64) pipeline. All chimeric tags were removed using the UCHIME algorithm (19), and finally obtained effective tags for further analysis. The tag sequence with the highest abundance was selected as the representative sequence within each cluster. The representative OTU sequences were classified into organisms by the Bayesian model using the RDP classifier (20) (version 2.2) based on the SILVA database (21). The abundance statistics of each taxonomic group were visualized using Krona (22) (version 2.6). The stacked bar plot of the community composition was visualized in the R project ggplot2 package (version 2.2.1). Circular layout representations of species abundance were graphed using Circos (version 0.69-3) (23). Heatmap of species abundance was plotted using the pheatmap package (version 1.0.12) in the R project.

Alpha diversity was applied to analyze the species diversity complexity of a sample through 6 indices: observed species, Chao1, Shannon, Simpson, ACE, and Good's coverage indices. All these indices in our samples were calculated with QIIME and displayed with R software (Version 2.2.1). Beta diversity analysis was used to evaluate the differences in the microbiota composition of the samples. Beta diversity was calculated with both weighted and unweighted UniFrac distances using QIIME software. To remove the sampling depth heterogeneity, rarefaction was performed to standardize the data obtained from samples with different sequencing efforts, and to compare the OTU richness of the samples using these standardized data (24). Rarefaction was used to randomly subsample the same number of sequences from each sample in order to compare the communities at a given level of sampling effort (an even sampling depth). Cluster analysis was preceded by principal coordinate analysis (PCoA), which was applied to reduce the dimensions of the original variables using the FactoMineR package and ggplot2 package in R software (Version 2.2.1). The ANOSIM non-parametric procedure (25) in the R project Vegan package (version 2.5.3) was used to test for significant differences among groups. Correlations between the differential bacterial genera and production traits and intestinal morphology parameters were investigated using Spearman's correlation analysis, with *p* < 0.05 was considered to indicate significant correlations.

### Statistical Analysis

Laying rate, egg quality trait and intestinal morphology parameters were subjected to analysis of variance (ANOVA) using the general linear model (GLM) command in SAS version 9.3 statistical software (SAS Institute Inc., Cary, NC, USA). Differences in the relative abundances of microbial community compositions between groups were analyzed with two-tailed non-parametric Mann-Whitney U test. All data are expressed as the means ± standard deviations (SDs). Differences were considered statistically significant at *p* < 0.05. The *p*-values were adjusted by the false discovery rate (FDR) using the Benjamini-Hochberg method.

## Results

### Production Performance

**Figure 1:**
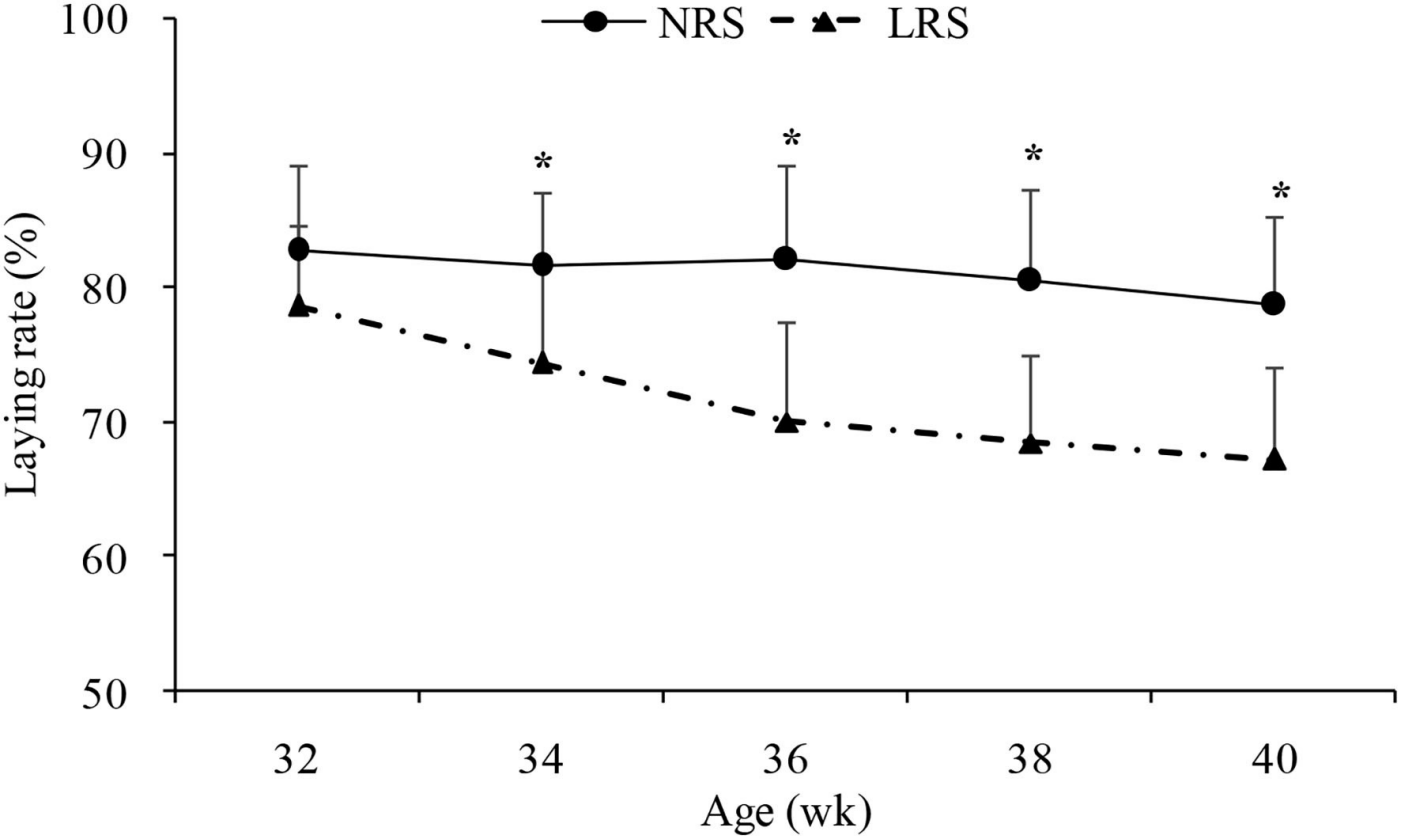
Laying rate of hens reared in the LRS and NRS from 32 to 40 weeks of age. LRS, floor litter housing system; NRS, plastic net housing system. Dotted lines represent the LRS; solid lines represent the NRS. *Means with asterisk superscripts within each period are significantly different (*p* < 0.05).

Production performance was measured by body weight, laying rate, mortality and some egg quality traits (Table 1). No significant difference in body weight or mortality rate was found between the two groups (*p* > 0.05). However, the laying rate in the NRS group decreased gradually (by 4.14%) with age, while there was a sharp decline in the LRS group (by 11.39%); the laying rate of birds in the NRS was significantly higher than that in birds in the LRS from 34 to 40 weeks of age (*p* < 0.05) (Figure 1). Egg quality traits of the two groups are shown in Supplementary Table 1. On average, egg weight and shell strength in the NRS group were slightly higher than those in the LRS group (*p* > 0.05), while the Haugh unit was slightly lower (*p* > 0.05).

**Table 1:** Production performance of laying hens reared in the LRS and NRS.

**Housing system^1^**	**Body weight, g**	**Laying rate, %**	**Mortality, %**	**Egg weight, g**	**Eggshell strength, kg/cm^**2**^**	**Haugh unit**
NRS	1,517.84 ± 140.62	81.69 ± 8.44^a^	0.04 ± 0.01	46.90 ± 1.46	4.43 ± 1.01	78.70 ± 9.01
LRS	1,522.96 ± 128.17	71.74 ± 8.81^b^	0.03 ± 0.01	46.15 ± 1.58	4.08 ± 1.13	80.07 ± 9.46

1 *NRS, plastic net housing system; LRS, floor litter housing system*.

a,b *Means with different superscripts within each column are significantly different (p < 0.05)*.

### Intestinal Morphology

The morphological parameters of the duodenum and cecum of laying hens reared in the LRS and NRS are shown in Table 2. A higher VH in the duodenum and a higher VCR in the cecum were observed in the NRS group than in the LRS group (*p* < 0.05).

**Table 2:** Intestinal morphological parameters of laying hens reared in the LRS and NRS.

**Intestinal parts**	**Item^1^**	**Housing system^2^**	
		**NRS, *n* = 20**	**LRS, *n* = 20**
Duodenum	VH	1,203.82 ± 157.49^a^	955.45 ± 164.07^b^
	CD	129.13 ± 14.92	111.61 ± 18.18
	VCR	9.29 ± 1.85	8.55 ± 1.63
Cecum	VH	605.80 ± 41.10	572.49 ± 31.90
	CD	101.83 ± 12.57	124.51 ± 13.04
	VCR	5.95 ± 0.79^a^	4.57 ± 0.67^b^

1 *VH, villus height; CD, crypt depth; VCR, villus height to crypt depth ratio*.

2 *NRS, plastic net housing system; LRS, floor litter housing system*.

a,b *Means with different superscripts within each row are significantly different (p < 0.05)*.

### Alpha Diversity Analysis

A total of 3,043,510 effective reads were obtained, including an average number of 81,873.89, 78,116.33, 90,437.11, and 87,740.44 for the LC (cecum of laying hens reared in the LRS), NC (cecum of laying hens reared in the NRS), LD (duodenum of laying hens reared in the LRS) and ND (duodenum of laying hens reared in the NRS) groups, respectively. The average length of the quality sequences for each sample was 435.59 reads. A total of 36,929 bacterial OTUs were detected, including an average number of 1,244.67, 1,188.33, 854.67, and 815.56 for the LC, NC, LD, and ND groups, respectively (Table 3). Indices of bacterial richness based on OTUs were estimated by the Ace and Chao methods, and indices of bacterial diversity were determined using the Simpson and Shannon methods (Table 3).

**Table 3:** Diversity estimation of the 16S rDNA gene libraries of the cecum and duodenum in laying hens reared on the LRS and NRS.

**Group**	**Effective reads**	**Average length**	**OTUs**	**Simpson**	**Shannon**	**ACE**	**Chao**	**Good's coverage**
LC, *n* = 10	81,873.89 ± 8,718.91	433.64 ± 30.73	1,244.67 ± 43.52^a^	0.99 ± 0.00	7.67 ± 0.18^a^	1,421.53 ± 62.25^a^	1,386.97 ± 63.01^a^	0.99 ± 0.00
NC, *n* = 10	78,116.33 ± 9,800.69	429.55 ± 35.75	1,188.33 ± 59.09^a^	0.98 ± 0.01	7.51 ± 0.24^a^	1,357.05 ± 68.20^a^	1,328.05 ± 62.56^a^	0.99 ± 0.00
LD, *n* = 10	90,437.11 ± 5,143.49	441.22 ± 41.46	854.67 ± 77.90^b^	0.83 ± 0.16	5.12 ± 0.70^b^	991.64 ± 84.41	986.73 ± 60.46^b^	0.99 ± 0.00
ND, *n* = 10	87,740.44 ± 9,487.34	437.92 ± 45.51	815.56 ± 66.37^b^	0.86 ± 0.15	4.90 ± 0.63^b^	941.91 ± 70.79	939.11 ± 74.72^b^	0.99 ± 0.00

*Operational taxonomic units (OTUs) were defined at 3% dissimilarity. Richness estimators (ACE and Chao) and diversity indices (Shannon) were calculated. LRS, floor litter housing system; NRS, plastic net housing system; LC, cecum of laying hens reared in the LRS; NC, cecum of laying hens reared in the NRS; LD, duodenum of laying hens reared in the LRS; ND, duodenum of laying hens reared in the NRS*.

a,b *Means with different superscripts within each column are significantly different (p < 0.05)*.

**Figure 2:**
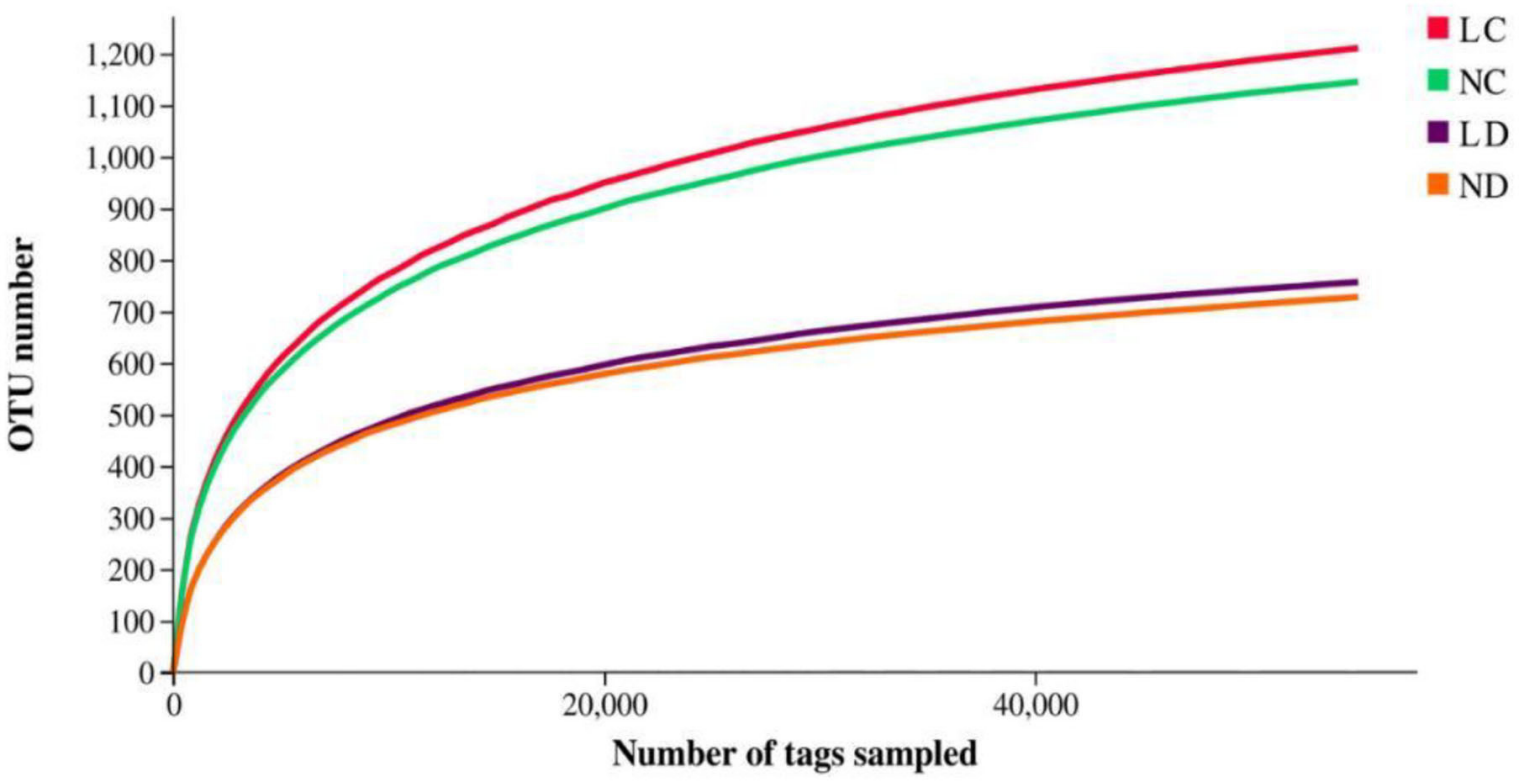
Rarefaction curves of LC, NC, LD and ND groups. LC, cecum of laying hens reared in the LRS; NC, cecum of laying hens reared in the NRS; LD, duodenum of laying hens reared in the LRS; ND, duodenum of laying hens reared in the NRS. LRS, floor litter housing system; NRS, plastic net housing system.

**Figure 3:**
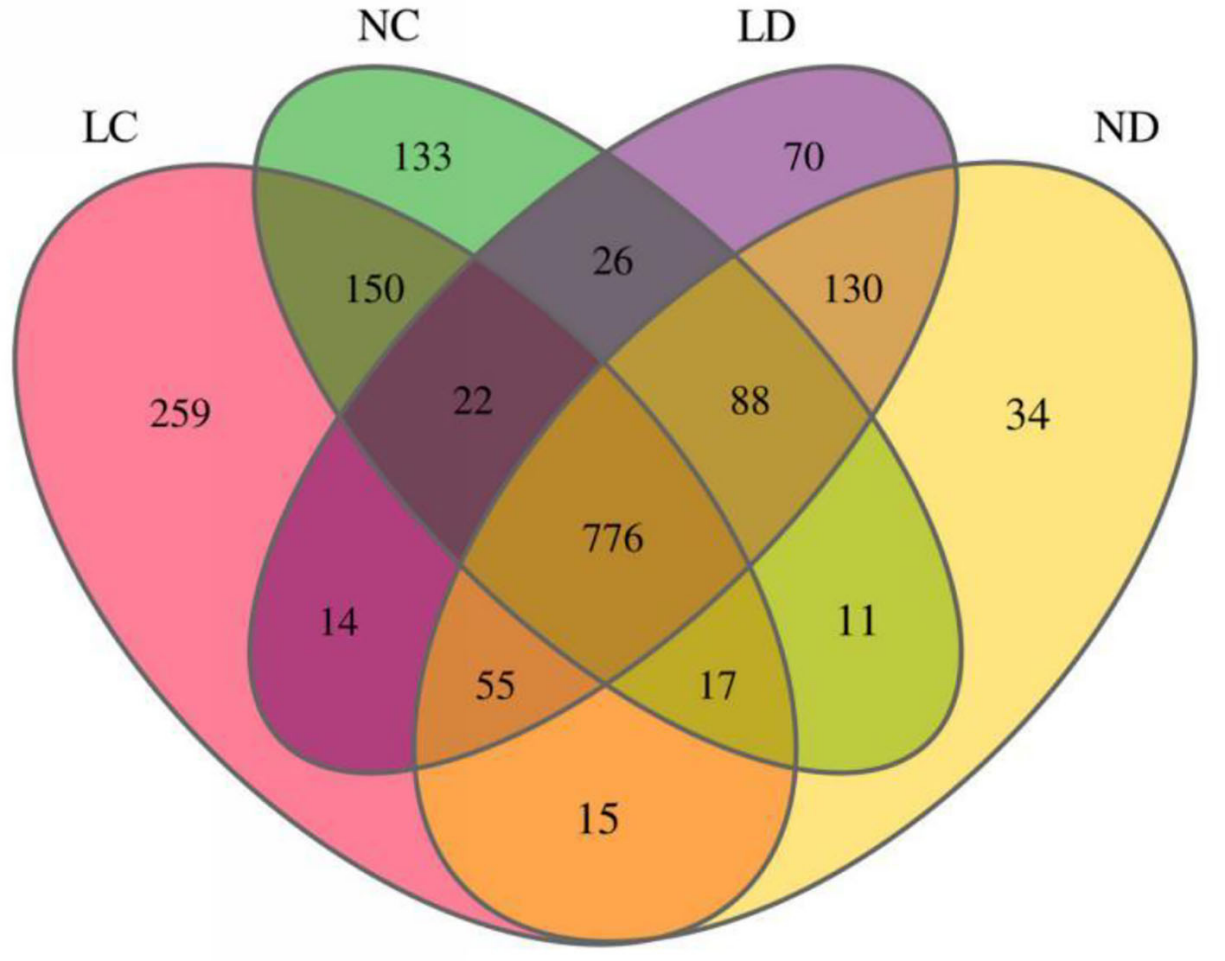
Flower plots of the cecal and duodenal microbiota of laying hens reared in the LRS and NRS (based on OTUs). Each circle in the Venn diagram represents one group noted by the same color. Numbers in the overlapping areas represent the number of bacterial OTUs shared between the respective groups. Numbers in the individual areas represent the number of bacterial OTUs exclusive to that group. LRS, floor litter housing system; NRS, plastic net housing system; LC, cecum of laying hens reared in the LRS; NC, cecum of laying hens reared in the NRS; LD, duodenum of laying hens reared in the LRS; ND, duodenum of laying hens reared in the NRS.

Rarefaction curves generated from the OTUs suggested that high sampling coverage was achieved in all groups (Figure 2). The cecal and duodenal contents included different numbers of bacterial OTUs under the different housing systems (Figure 3). The LC and NC groups shared 965 bacterial OTUs, while 341 bacterial OTUs were uniquely sequenced in the LC group compared with 258 in the NC group. The LD and ND groups shared 1,049 bacterial OTUs, while 132 bacterial OTUs were uniquely sequenced in LD, compared with 77 in ND. The observed bacterial OTUs and Shannon indices in the cecum and duodenum were significantly higher in laying hens reared in the LRS than in those reared in the NRS (*p* < 0.05).

### Beta Diversity Analysis

**Figure 4:**
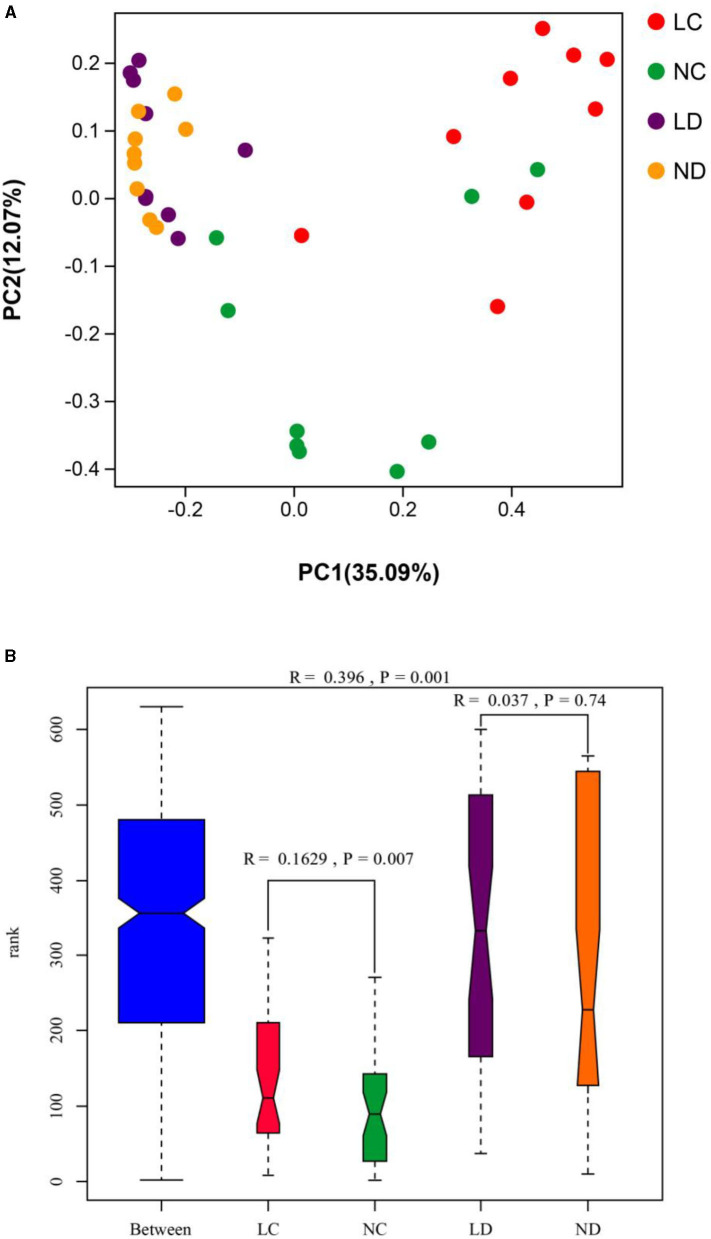
**(A)** PCoA (based on the unweighted UniFrac distance) and **(B)** ANOSIM of cecal and duodenal microbiota in laying hens reared in the LRS and NRS. PC1 and PC2 on the x- and y-axes represent two principal discrepancy components among groups, and the percentage in brackets indicates the contribution to the discrepancy component. Dots represent samples. Samples in the same group share the same color. LRS, floor litter housing system; NRS, plastic net housing system; LC, cecum of laying hens reared in the LRS; NC, cecum of laying hens reared in the NRS; LD, duodenum of laying hens reared in the LRS; ND, duodenum of laying hens reared in the NRS.

PCoA was conducted using sample distance matrices generated based on their group species-level phylogenetic and evolutionary relationships. In the unweighted UniFrac PCoA, the first principal coordinate (PC1) explained 35.09% of the variation among samples, and PC2 explained 12.07% of the variation (Figure 4A). The sample dots from different intestinal tracts (cecum and duodenum) showed distinct distances, with long distances between LC and NC, while there were no differences in distance between LD and ND. Comparisons of the cecal and duodenal microbiota compositions between LRS and NRS using ANOSIM (Figure 4B) showed significant differentiation in cecum (*R* = 0.163, *p* = 0.007), and non-significant differentiation in the duodenum (*R* = 0.037, *p* = 0.74).

### Taxonomic Composition of the Duodenum and Cecum Between the Two Housing Systems

**Figure 5:**
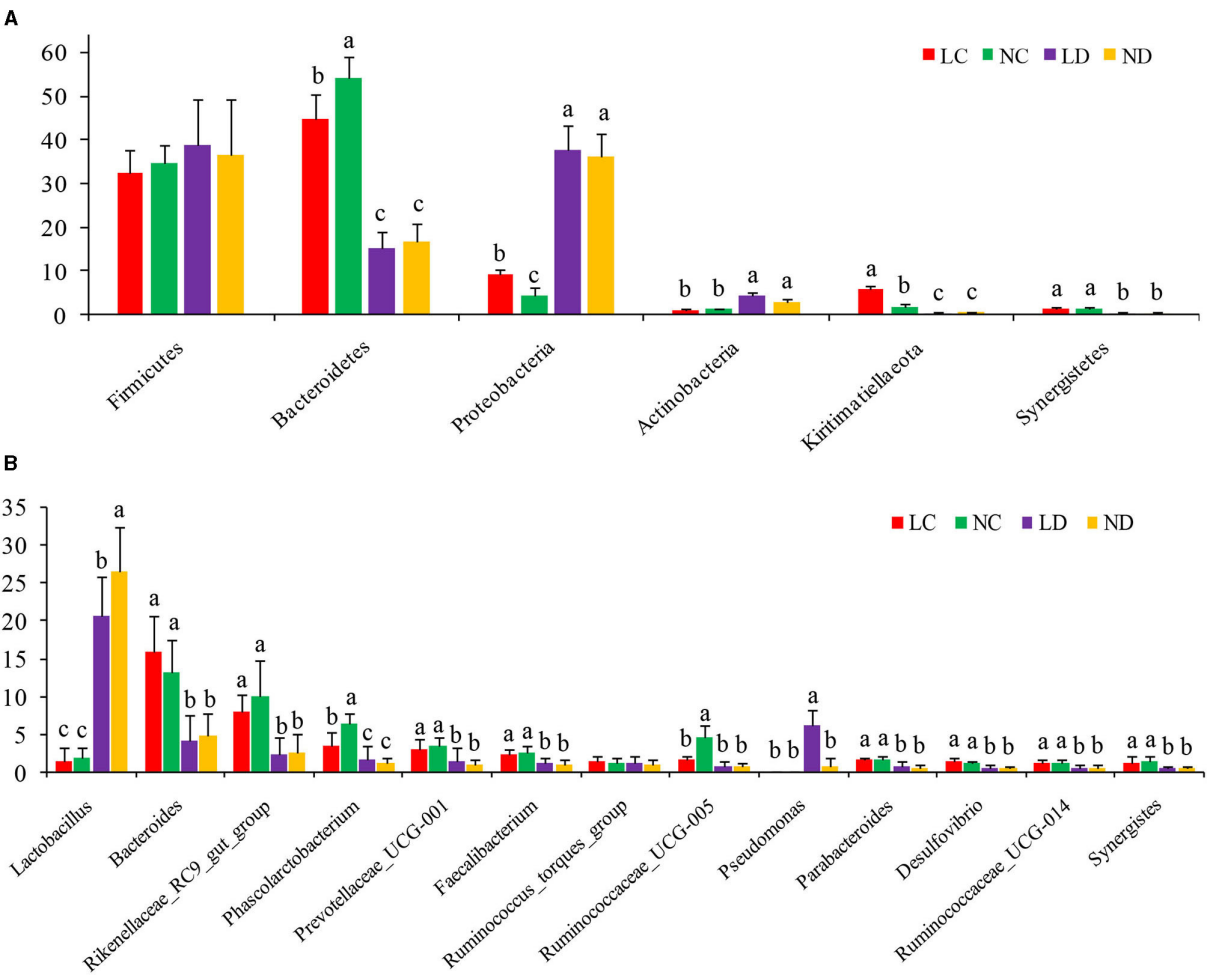
The relative abundance (% reads) of **(A)** the most dominant phylum and **(B)** the most dominant genus in the cecal and duodenal microbiome of laying hens reared in the LRS and NRS. Error bars represent the SD of samples. Boxes with a different letter above the error bars are significantly different at *p* < 0.05 by *t*-test analyses. LRS, floor litter housing system; NRS, plastic net housing system; LC, cecum of laying hens reared in the LRS; NC, cecum of laying hens reared in the NRS; LD, duodenum of laying hens reared in the LRS; ND, duodenum of laying hens reared in the NRS.

**Figure 6:**
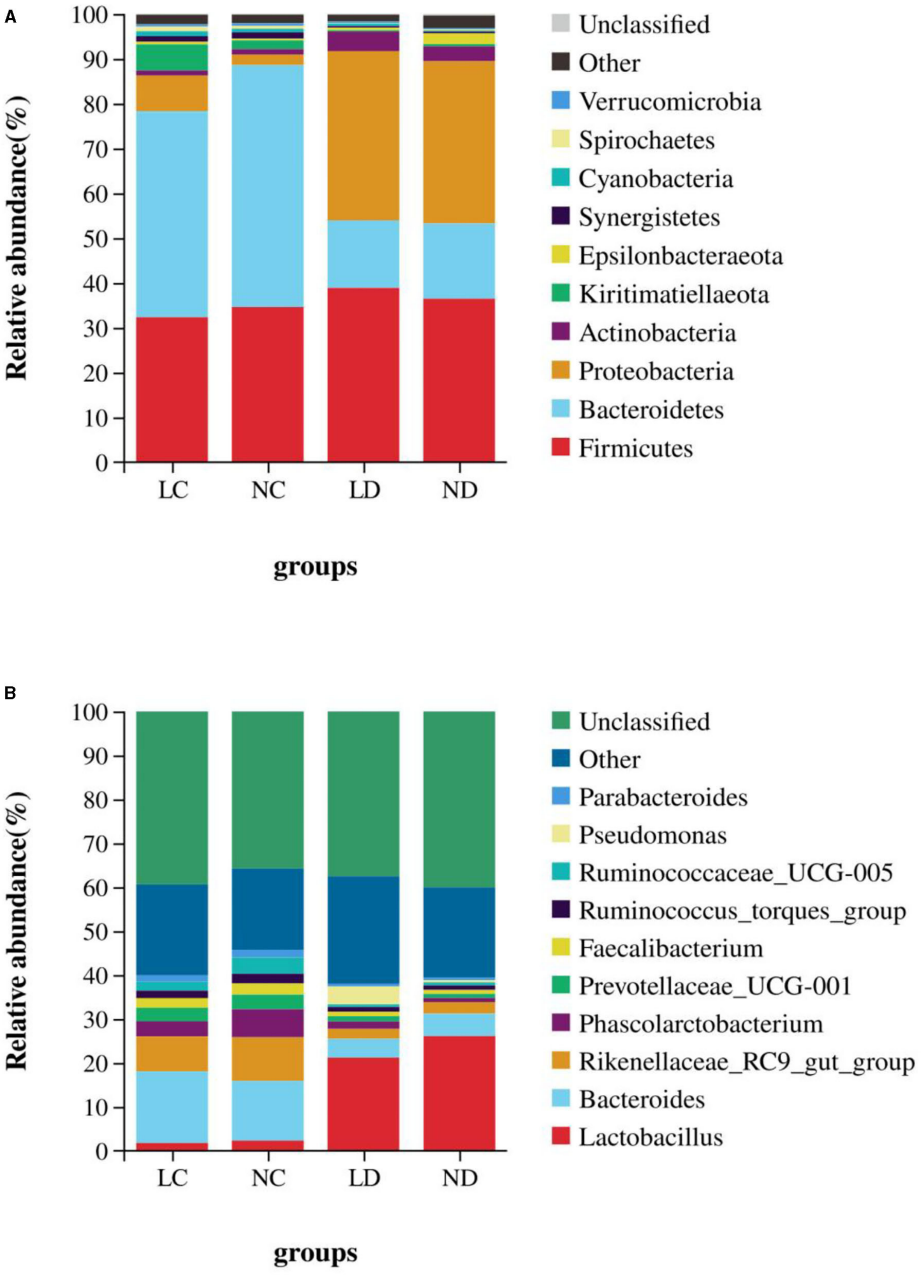
The relative abundance of cecal and duodenal bacteria at the phylum **(A)** and genus **(B)** levels in laying hens reared in the LRS and NRS. LRS, floor litter housing system; NRS, plastic net housing system; LC, cecum of laying hens reared in the LRS; NC, cecum of laying hens reared in the NRS; LD, duodenum of laying hens reared in the LRS; ND, duodenum of laying hens reared in the NRS.

Based on the SILVA taxonomic database and using the analysis function of the RDP Classifier14, all sequences were classified from phylum to species. A total of 26 different phyla were detected in these samples. The four groups showed dissimilar taxonomic compositions at the phylum level (Figures 5A, 6A; Supplementary Table 2). Within the cecum, Firmicutes (32.36%), Bacteroidetes (44.70%), Proteobacteria (9.18%) and Kiritimatiellaeota (5.81%) were the predominant phyla for LC, and Firmicutes (34.67%), Bacteroidetes (54.01%), and Proteobacteria (4.29%) were the predominant phyla for NC. Within the duodenum, LD and ND were both predominated by Firmicutes, Bacteroidetes, Proteobacteria and Actinobacteria, which represented 38.89, 15.06, 37.77, and 4.36% of the total reads for LD and 36.48, 16.78, 36.19, and 3.11% of the total reads for ND. The abundances of Bacteroidetes, Proteobacteria, Actinobacteria, Kiritimatiellaeota, and Synergistetes differed significantly between the cecum and duodenum in both housing systems.

Detected sequences were assigned to 360 different genera, and the relatively most abundant bacteria are shown in Figures 5B, 6B; Supplementary Table 3. Within the cecum, the relatively most abundant bacteria were *Lactobacillus, Bacteroides, Rikenellaceae_RC9_gut_group, Phascolarctobacterium, Prevotellaceae_UCG-001, Faecalibacterium, Ruminococcus_torques_group, Ruminococcaceae_UCG-005, Parabacteroides, Desulfovibrio, Ruminococcaceae_UCG-014*, and *Synergistes*. Within the duodenum, the relatively most abundant bacteria were *Lactobacillus, Bacteroides, Rikenellaceae_RC9_gut_group, Phascolarctobacterium, Prevotellaceae_UCG-001, Faecalibacterium, Ruminococcus_torques_group*, and *Parabacteroides*. The abundances of most bacterial genera were differentially detected between the cecum and duodenum in both housing systems (*p* < 0.05).

### Differences in Bacterial Communities Between Housing Systems

Figure 5A shows that the microbial compositions of the cecum and duodenum at the phylum level differed between the LRS and NRS. Within the cecum, Proteobacteria and Kiritimatiellaeota were significantly more abundant in the LRS than in the NRS (*p* < 0.05), while Bacteroidetes were significantly less abundant in the LRS (*p* < 0.05). Within the duodenum, there were no significant differences in the dominant bacterial phyla between the two housing systems (*p* > 0.05).

Figure 5B shows that the microbial compositions of the cecum and duodenum at the genus level differed between the LRS and NRS. Within the cecum, *Phascolarctobacterium* and *Ruminococcaceae_UCG-005* were significantly less abundant in the LRS (*p* < 0.05) than in the NRS, while the relative abundance of *Bacteroides* was slightly higher in the LRS. Within the duodenum, *Lactobacillus* was significantly less abundant in the LRS (*p* < 0.05) than in the NRS, while *Pseudomonas* was significantly more abundant in the LRS (*p* < 0.05). There were no significant differences in other dominant genera in the cecum or in the duodenum between the two housing systems.

### Correlation Analysis of Differentially Detected Bacterial Genera With Production Performance and Intestinal Morphological Parameters

Spearman's correlation analysis was performed based on the relative abundance of the above differential bacterial genera and production performance and intestinal morphological parameters (Table 4). Cecal *Phascolarctobacterium* was significantly positively correlated with eggshell strength (*R* = 0.608, *p* < 0.01). Cecal *Ruminococcaceae_UCG-005* was significantly positively correlated with egg weight (*R* = 0.526, *p* < 0.05). Duodenal *Lactobacillus* was significantly positively correlated with VH and VCR (*R* = 0.548 and 0.565, *p* < 0.05). Duodenal *Pseudomonas* was significantly negatively correlated with the Haugh units (*R* = −0.550, *p* < 0.05).

**Table 4:** Correlations of differentially detected bacterial genera with production performance and intestinal morphological parameters in the two housing systems.

**Intestinal part**	**Genus**	**Laying**	**Egg**	**Eggshell**	**Haugh**	**VH**	**CD**	**VCR**
		**rate**	**weight**	**strength**	**units**			
Cecum	*Phascolarctobacterium*	−0.038	−0.021	0.608^**^	−0.036	−0.020	−0.031	0.370
	*Ruminococcaceae_UCG-005*	−0.218	0.526^*^	0.018	−0.044	0.342	−0.389	0.220
Duodenum	*Lactobacillus*	0.053	0.417	0.226	0.304	0.548^*^	−0.406	0.565^*^
	*Pseudomonas*	−0.126	−0.093	−0.053	−0.550^*^	−0.238	0.275	−0.363

*VH, villus height; CD, crypt depth; VCR, villus height to crypt depth ratio*.

* *p < 0.05;*

** *p < 0.01*.

## Discussion

Housing systems in poultry houses have been the focus of scientific research for many years and are an external factor influencing both bird growth and health. The current results revealed that housing systems could affect the production traits of laying hens, although not all indicators were significantly influenced. As reported by Dong et al. (26) in Xianju chickens, rearing systems had significant effects on egg production but had negligible effects on egg quality traits. Similarly, there was no difference in egg weight, shell strength or HUs between the two housing systems in the present study, and lower egg production in the LRS indicated negative laying performance. A larger VH and VCR in the duodenum as well as a larger VCR in the cecum were observed in the NRS group than in the LRS group. These findings were similar to the results of Li et al. (27), who found that broilers reared in the NRS had higher jejunal VH and VCR, with lower CD than broilers reared in the LRS. Compared with the LRS groups, laying hens in the NRS groups were fed on perforated plastic nets with no direct contact with feces, which may have accounted for the changes in the intestinal mucosal structure.

Duodenum locates at the beginning of the small intestine, which has a lower pH than the hindgut and is crucial for feed digestion and absorption (5). Cecum is the chief functional section in the distal intestine, which plays important roles in preventing pathogen colonization, detoxifying harmful substances and absorbing additional nutrients (7). Although the cecum and the duodenum are important for laying hens, the effects of different housing conditions on its microbial communities have not been studied extensively. In this study, we subjected laying hens to different non-cage housing systems (LRS and NRS) and kept other factors constant to examine their effects on the ceecal and duodenal microbiota. The results revealed that housing conditions, irrespective of nutritional formulation and other factors, affected the composition of cecal and duodenal microbiota in hens by 16S rRNA profiling. The diversity of the cecal and duodenal microbiota was greater in the LRS than in the NRS (Figures 2, 3), as indicated by higher bacterial OTUs and Shannon indices. At the phylum level, the dominant bacterial phyla in the cecum were Bacteroidetes, Firmicutes, and Proteobacteria, which were similar to the dominant bacterial phyla in the duodenum comprising Bacteroidetes, Firmicutes, Proteobacteria, and Actinobacteria. These results supported the findings of other studies (28) showing that have shown that chicken intestinal microbes are dominated by Firmicutes and Bacteroidetes, which are also commonly observed in the gut environments of other birds (29). Interestingly, the abundances of Proteobacteria and Kiritimatiellaeota in the cecum were significantly higher in the LRS, while the abundance of Bacteroidetes in the cecum was significantly higher in the NRS (Figure 5A), whereas these phyla showed no difference in the duodenum between the two housing systems. The role of Proteobacteria in apparent nutrient digestion has been frequently been reported to be associated with cellulose activity (30), while Kiritimatiellaeota is a newly described phylum, and little is known about its members. Spring et al. (31) showed that Kiritimatiella glycovorans L21-Fru-ABT fermented xylose to ethanol and acetate but was not able to utilize starch, sucrose, or fructose. The differential abundance of cecal Proteobacteria and Kiritimatiellaeota in our study could be related to the housing conditions. Bacteroidetes are involved in many metabolic activities, including the fermentation of carbohydrates, utilization of nitrogenous substances, and maintenance of intestinal microecological balance (32, 33). Therefore, the superior cecal morphology (higher VCR) in the NRS may also be related to the higher abundance of Bacteroidetes.

At the genus level, the abundance of *Phascolarctobacterium* and *Ruminococcaceae_UCG-005* in cecum and *Lactobacillus* in duodenum was higher in NRS, while the abudance of *Pseudomonas* in duodenum was higher in LRS (Figure 5B). The differences in cecal and duodenal microbes between the housing systems are likely to be a result of differences in circumstances and environmental pressure. *Phascolarctobacterium* can produce short-chain fatty acids, including acetate and propionate, and it can be associated with the metabolic state and mood of the host (34). *Ruminococcaceae* are regarded as potential beneficial bacteria because they participate in the positive regulation of the intestinal environment and are linked to immunomodulation and healthy homeostasis (35). *Lactobacillus* is widely known as a probiotic in the guts of animals and humans and is beneficial for digestion and immunity (36). A higher abundance of these genera in the NRS exhibited the better health status of hens, indicating advantages of the NRS and could be conducive to the production performance and the repair of the intestinal mucosa, possibly contributing to a higher laying rate and VCR than those in the LRS (Tables 1, 2).

Correlation analysis showed that the abundance of differential bacterial genera was significantly correlated with some production traits and intestinal morphological parameters. *Phascolarctobacterium* has been reported to play an important role in the metabolic pathways of chickens, complementing the absence of carbohydrate metabolism by an increase in lipid metabolism, with methylmalonyl-CoA mutase and methylmalonyl-CoA carboxyltransferase being the most abundant lipid metabolism proteins (37). *Phascolarctobacterium* was significantly positively correlated with eggshell strength, which is similar to the results of Gan et al. (38), who found that cecal *Phascolarctobacterium* was correlated with the production performance and egg quality of aged laying hens. The existence of *Phascolarctobacterium* in the cecum may be related to the deposition of calcium and magnesium in the uterus (shell gland) of the oviduct. Members of *Ruminococcaceae* have been reported to be associated with growth performance in broilers (39) and are highly abundant in birds with efficient feed conversion (40). They can be an indicator of feed efficiency in cecal digesta (41). Similarly, a significant positive correlation between the abundance of *Ruminococcaceae_UCG-005* and egg weight was found in the present study, which showed that this organism may help improve the feed conversion ratio of laying hens.

*Lactobacillus* can exert their beneficial effects on the intestinal mucosa by improving the microflora balance and inhibiting the colonization of pathogens (42). The small intestine is the main organ in the gastrointestinal tract, and its structure supports the digestion and absorption of nutrients such as VH, CD, and VCR, which can reflect the functional status of the small intestine (43, 44). In the present study, *Lactobacillus* was significantly positively correlated with VH and the VCR in the duodenum, indicating that hens with more *Lactobacillus* in the duodenum may have an improved intestinal morphology and increased digestion and absorption capacity. Accordingly, hens in the NRS with a higher abundance of Lactobacillus had a higher duodenal VH and VCR than those in the LRS. The finding was similar to the results of Cui and Xu (45), who reported that a higher percentage of *Lactobacillus* in the chicken ileum might result in more expressive anti-inflammatory factors. Similarly, Chae reported that dietary supplementation with probiotic *Lactobacillus acidophilus* could increase the VH in the duodenum and the VCR in the ileum of broilers (46). *Pseudomonas* is one of the most complex bacterial genera with the largest number of known species of which many species are pathogenic to plants and a few are pathogenic to animals (47, 48). Although the differential abundance of *Pseudomonas* between the two housing groups did not result in a significant difference in the Haugh unit in the present study, it is notable that the abundance of *Pseudomonas* in the duodenum was significantly negatively correlated with the Haugh unit, indicating a tendency for these bacteria to have adverse effects on egg quality traits. Thus, it is reasonable to hypothesize that the increase in *Pseudomonas* in the intestine may degrade the thick albumen secretion in the magnum of laying hens.

In conclusion, there were differences in the cecal and duodenal microbiota compositions of Shendan chickens reared in different non-cage housing systems (NRS and LRS). The abundances of differentially detected bacterial genera *Phascolarctobacterium, Ruminococcaceae_UCG-005, Lactobacillus* and *Pseudomonas* were significantly correlated with some egg quality traits and intestinal morphology parameters. The current findings provided support for the advantages of the NRS in improving the laying performance and intestinal morphology and microecological environment of Shendan chickens.

## Data Availability Statement

The raw data of 16S rRNA gene presented in the study are deposited in NCBI repository, accession number PRJNA743133.

## Ethics Statement

The experimental protocol of the current study was approved by the Committee for the Care and Use of Experimental Animals at Anhui Academy of Agricultural Science under permit No. A11-CS06.

## Author Contributions

YW and KZ conceived and designed the study. RM, HZ, and LL performed the experiments. YW and LC wrote the original paper. JL, WL, and YL analyzed the sequencing data and experimental results. KZ obtained project funding and edited the manuscript. All authors have read and agreed to the published version of the manuscript.

## Funding

This work was supported by the China Agriculture Research System of MOF and MARA (Grant No. CARS-40-K21), the Nature Science Foundation of Anhui Province (Grant No. 1908085QC115), and the Major Science and Technology Project of Anhui Province (Grant No. 201903a06020020).

## Conflict of Interest

HZ and LL were employed by the company Hubei Shendan Health Food Co., Ltd. The remaining authors declare that the research was conducted in the absence of any commercial or financial relationships that could be construed as a potential conflict of interest.

## Publisher's Note

All claims expressed in this article are solely those of the authors and do not necessarily represent those of their affiliated organizations, or those of the publisher, the editors and the reviewers. Any product that may be evaluated in this article, or claim that may be made by its manufacturer, is not guaranteed or endorsed by the publisher.
