# Supplementary material for: Different Non-cage Housing Systems Alter Duodenal and Cecal Microbiota Composition in Shendan Chickens
Source: Front Vet Sci. 2021 Oct 6;8:728538. doi: 10.3389/fvets.2021.728538 (PMC8526545; doi:10.3389/fvets.2021.728538)
Supplement: Supplementary file 1 [file Table_1.docx]

Supplementary Material

# Supplementary Tables

**Supplementary Table 1.** **Egg quality traits of hens reared in the LRS and NRS from 32 to 40 weeks of age.** LRS, floor litter housing system; NRS, plastic net housing system. ^a,b^Means with different superscripts within each row are significantly different (p < 0.05)

| Age, week | Egg weight, g | | Eggshell Strength, kg/cm^2^ | | Haugh Unit | |
| --- | --- | --- | --- | --- | --- | --- |
|  | NRS | LRS | NRS | LRS | NRS | LRS |
| 32 | 46.26±2.01 | 45.96±1.67 | 5.15±1.11^a^ | 4.97±0.96^a^ | 83.65±8.83 | 82.80±9.97 |
| 34 | 46.98±1.67 | 46.25±1.44 | 3.69±0.90^b^ | 4.19±1.34^ab^ | 75.02±7.72 | 80.48±8.66 |
| 36 | 47.34±1.15 | 46.09±2.17 | 4.73±1.27^ab^ | 3.72±1.25^b^ | 80.31±9.29 | 79.22±9.72 |
| 38 | 47.21±1.24 | 46.27±1.21 | 4.96±1.02^a^ | 3.64±0.83^b^ | 76.21±9.11 | 76.16±8.93 |
| 40 | 47.39±1.32 | 46.42±1.48 | 3.81±0.91^b^ | 4.05±1.47^ab^ | 78.80±8.64 | 82.69±9.25 |
| Mean | 46.90±1.46 | 46.15±1.58 | 4.43±1.01^ab^ | 4.08±1.13^ab^ | 78.70±9.01 | 80.07±9.46 |

**Supplementary Table 2.** **The relative abundance (% reads) of the most dominant phyla in the cecal and duodenal microbiome of layers reared on LRS and NRS.** LRS, floor litter housing system; NRS, plastic net housing system; LC, cecum of layers reared on LRS; NC, cecum of layers reared on NRS; LD, duodenum of layers reared on LRS; ND, duodenum of layers reared on NRS. ^a–c^Means with different superscripts in the same row are significantly different (p < 0.05)

| Species | LC | NC | LD | ND |
| --- | --- | --- | --- | --- |
| Firmicutes | 32.36±5.13 | 34.63±4.20 | 38.89±10.24 | 36.48±12.71 |
| Bacteroidetes | 44.70±5.65^b^ | 54.01±4.76^a^ | 15.06±3.71^c^ | 16.78±4.00^c^ |
| Proteobacteria | 9.18±1.31^b^ | 4.29±1.76^c^ | 37.77±5.62^a^ | 36.19±5.32^a^ |
| Actinobacteria | 1.56±0.20^b^ | 1.23±0.22^b^ | 4.36±0.87^a^ | 3.11±0.73^a^ |
| Kiritimatiellaeota | 5.81±0.88^a^ | 1.94±0.74^b^ | 0.29±0.19^c^ | 0.50±0.18^c^ |
| Synergistetes | 1.25±0.38^a^ | 1.44±0.36^a^ | 0.37±0.14^b^ | 0.39±0.10^b^ |

**Supplementary Table 3. The relative abundance (% reads) of the most dominant genera in the cecal and duodenal microbiome of layers reared on LRS and NRS.** LRS, floor litter housing system; NRS, plastic net housing system; LC, cecum of layers reared on LRS; NC, cecum of layers reared on NRS; LD, duodenum of layers reared on LRS; ND, duodenum of layers reared on NRS. ^a-c^Means with different superscripts in the same row are significantly different (p < 0.05).

| Species | LC | NC | LD | ND |
| --- | --- | --- | --- | --- |
| *Lactobacillus* | 1.32±1.93^c^ | 1.79±1.49c | 20.70±5.08b | 26.50±5.88^a^ |
| *Bacteroides* | 15.76±4.83^a^ | 13.11±4.34^a^ | 4.22±3.35^b^ | 4.89±2.87^b^ |
| *Rikenellaceae_RC9_gut_group* | 7.94±2.36^a^ | 9.93±4.91^a^ | 2.28±2.19^b^ | 2.62±2.45^b^ |
| *Phascolarctobacterium* | 3.47±1.82^b^ | 6.39±1.25^a^ | 1.71±1.83^c^ | 1.19±0.58^c^ |
| *Prevotellaceae_UCG-001* | 3.07±1.21^a^ | 3.33±1.19^a^ | 1.38±1.78^b^ | 0.95±0.70^b^ |
| *Faecalibacterium* | 2.20±0.74^a^ | 2.54±0.86^a^ | 1.14±0.68^b^ | 0.89±0.67^b^ |
| *Ruminococcus_torques_group* | 1.50±0.56 | 1.22±0.63 | 1.15±0.90 | 1.03±0.70 |
| *Ruminococcaceae_UCG-005* | 1.52±0.54^b^ | 4.67±1.43^a^ | 0.80±0.52^b^ | 0.69±0.56^b^ |
| *Pseudomonas* | 0.01±0.02^b^ | 0.01±0.02^b^ | 6.07±2.14^a^ | 0.69±1.15^b^ |
| *Parabacteroides* | 1.52±0.27^a^ | 1.60±0.54^a^ | 0.77±0.53^b^ | 0.56±0.40^b^ |
| *Desulfovibrio* | 1.47±0.47^a^ | 1.18±0.32^a^ | 0.49±0.36^b^ | 0.49±0.35^b^ |
| *Ruminococcaceae_UCG-014* | 1.15±0.54^a^ | 1.26±0.40^a^ | 0.54±0.47^b^ | 0.59±0.29^b^ |
| *Synergistes* | 1.25±0.78^a^ | 1.44±0.56^a^ | 0.47±0.34^b^ | 0.39±0.39^b^ |
